# Comparison of Subdural and Intracortical Recordings of Somatosensory Evoked Responses

**DOI:** 10.3390/s24216847

**Published:** 2024-10-25

**Authors:** Felipe Rettore Andreis, Suzan Meijs, Thomas Gomes Nørgaard dos Santos Nielsen, Taha Al Muhamadee Janjua, Winnie Jensen

**Affiliations:** Center for Neuroplasticity and Pain, Department of Health Science and Technology, Aalborg University, Selma Lagerløfs Vej 249, 9260 Aalborg, Denmark; smeijs@hst.aau.dk (S.M.); thnn@hst.aau.dk (T.G.N.d.S.N.); taha@hst.aau.dk (T.A.M.J.); wj@hst.aau.dk (W.J.)

**Keywords:** µECoG, intracortical microelectrode array, somatosensory evoked potentials, primary somatosensory cortex

## Abstract

Micro-electrocorticography (µECoG) electrodes have emerged to balance the trade-off between invasiveness and signal quality in brain recordings. However, its large-scale applicability is still hindered by a lack of comparative studies assessing the relationship between ECoG and traditional recording methods such as penetrating electrodes. This study aimed to compare somatosensory evoked potentials (SEPs) through the lenses of a µECoG and an intracortical microelectrode array (MEA). The electrodes were implanted in the pig’s primary somatosensory cortex, while SEPs were generated by applying electrical stimulation to the ulnar nerve. The SEP amplitude, signal-to-noise ratio (SNR), power spectral density (PSD), and correlation structure were analysed. Overall, SEPs resulting from MEA recordings had higher amplitudes and contained significantly more spectral power, especially at higher frequencies. However, the SNRs were similar between the interfaces. These results demonstrate the feasibility of using µECoG to decode SEPs with wide-range applications in physiology monitoring and brain–computer interfaces.

## 1. Introduction

The study of brain activity is a fundamental pursuit in neuroscience and neuro-engineering, aiming to unravel the complexities of cognition, behaviour, and neurological disorders [1]. Over the years, significant advances in electrophysiological techniques have transformed our ability to investigate the brain’s electrical activity, progressing from single-neuron recordings to the utilisation of penetrating high-density microelectrode arrays (MEAs) and ultra-flexible electrocorticography (ECoG) arrays [2].

Neural signals recorded from the extracellular space reveal the presence of two distinct voltage signal types: action potentials or spikes, which are brief, millisecond-scale events originating from individual neurons, and slow-varying local field potentials (LFPs) representing the aggregate transmembrane currents generated by multiple neurons [2]. Intracortical MEAs implanted directly into the cerebral cortex offer high spatial resolution and the ability to capture neural responses from deep cortical layers. However, the insertion of MEAs into the brain causes significant damage to the surrounding tissue, ultimately hindering chronic stability and the recording performance [3].

Growing interest has arisen in ECoG electrodes placed on the surface of the cerebral cortex either epidurally or subdurally [4], aiming to provide an optimal compromise between invasiveness and recording performance. Traditional ECoGs consist of electrode diameters of ~5 mm with 5–10 mm interelectrode distance [5]. The relatively large electrodes are more prone to source cancellation by the destructive interference from neuronal subpopulations and result in the blurring of spatial topography within cortical regions [5]. The limitations of traditional ECoG have led researchers to develop a new generation of ECoGs that contain smaller electrode diameters (<250 µm) and interelectrode spacing (<1.5 mm). Signals recorded with micro-ECoG (µECoG) contain a broader spectral band power, and the signals contain information to decode spatially confined neural populations with stable recordings over months [6]. In addition, µECoG signals have been shown to approximate a better penetrating microelectrode array (MEA) in the temporal and spatial scales [7] and improve the prediction of, e.g., seizure outcomes compared to traditional ECoG [8]. Another advantage is the use of ultra-flexible materials that allow conforming to the shape of the cortical surface, improving the electrode–tissue contact.

Direct comparisons between penetrating MEAs and µECoGs have been previously performed to characterise the contribution of LFPs at different cortical depths to the electrocorticogram signal in a reach-and-grasp task [9]; the authors showed that ECoG signals are similar to LFPs close to the surface, particularly for low-frequency bands. LFPs have also been compared to µECoG signals in natural behaviour (i.e., spontaneous brain activity), and it was observed that the association between these signals tended to decline gradually as a function of space, time, and frequency [7]. In motor-related tasks and especially spontaneous brain activity, inputs are internally generated, so variability cannot be estimated. This limitation can be overcome using controlled external stimuli and measuring from sensory-related areas [10] so that signal features can be largely accounted for by electrode characteristics. For evoked potentials, however, comparisons are limited for MEA and traditional-size ECoGs [10,11,12]. Therefore, in this study, we aimed to provide a comparative analysis of intracortical MEA and subdural µECoG using somatosensory evoked potentials (SEPs) in the pig’s primary somatosensory cortex (S1).

## 2. Materials and Methods

Twenty healthy female Danish Landrace pigs (38.4 ± 10.7 kg) were included in this study. All procedures were in accordance with the Danish Veterinary and Food Administration under the Ministry of Environment and Food of Denmark (protocol number: 2017-15-0201-0137).

### 2.1. Surgical Procedures

The animals were first tranquilised in their home pen with a blend (of 5 mL zoletil, 25 mg/mL tiletamine, 25 mg/mL zolazepam, 6.25 mL xylazine (20 mg/mL), 1.25 mL ketamine (100 mg/mL), and 2.5 mL butorphanol (10 mg/mL)) and moved to the surgical room. After that, the animals were placed in a prone position and intubated. Anaesthesia was maintained during surgery with sevoflurane (1.5–2.5% minimum alveolar concentration), propofol (2 mg/h/kg), and fentanyl (10 μg/h/kg). After the surgery and before the recordings were initiated, sevoflurane was gradually decreased to zero since it is a known suppressor of somatosensory evoked potential [13].

Vital functions and depth of anaesthesia were assessed by ECG, end-tidal CO_2_, blood pressure, and respiratory rate. Body temperature was kept between 36 and 38 °C using a heated surgical table pad (Mistral-Air^®^ MA1100, Amersfoort, The Netherlands).

The animal’s head was fixated using a custom-designed stereotaxic frame affixed to their heads with screws anchored in the zygomatic arch. A craniotomy procedure was carried out, creating a rectangular opening measuring three by five centimetres centred around the bregma landmark. Adjacent to this rectangle, two holes were carefully drilled to accommodate stainless steel screws used for grounding and referencing purposes. Subsequently, surgical scissors and micro forceps were employed to remove the dura matter while avoiding any disruption of blood vessels on the exposed cortex. The primary somatosensory cortex (S1) and its respective forelimb representation were located following [14]. More details on the surgical procedure can be obtained in a previous publication [15].

### 2.2. Electrode Specification and Data Recording

Nine animals were implanted with an intracortical MEA (Model #MEA-PI-A3-00-16-0.6-2.0-3-1.0-1.0-1-1SS-1, Microprobes Inc., Gaithersburg, MD, USA) with Pt/Ir electrodes. The squared grid array contained 16 electrodes (4 × 4) with biocompatible Parylene-C isolation except at the tip. The MEA had an interelectrode distance of 1.0 mm. The electrode length below the epoxy was 2 mm, and the tip diameter was 75 μm with an impedance of 0.6 MΩ. The MEA was penetrated 1.6–1.8 mm perpendicularly into the cortex using a micromanipulator.

**Figure 1:**
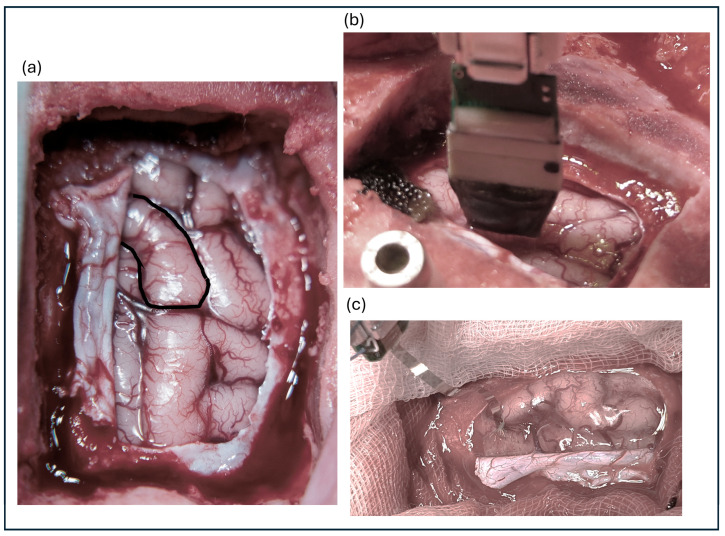
(**a**) Exposed cortical surface with the primary somatosensory cortex highlighted. (**b**) Implanted microelectrode array positioned on the cortex. (**c**) Close-up view of the microelectrocorticography (µECoG) array.

Eleven animals were subdurally implanted with a µECoG (Model #E32-1000-30-200-HZ32, Neuronexus Technologies, Ann Arbor, MI, USA). The array consisted of 32 electrodes with diameters of 200 µm and impedance values ranging from 10 to 40 kΩ. The electrode was arranged in an 8 × 4 configuration with an interelectrode distance of 1 mm. Figure 1a shows the cortex view with S1 highlighted, whereas Figure 1b shows the implanted MEA and Figure 1c shows the µECoG.

The recordings started one hour after electrode implantation to allow tissue responses and impedances to stabilise, and the cortex was periodically flushed with isotonic saline.

To produce somatosensory evoked potentials (SEPs), two cuff electrodes were implanted around the pig’s ulnar nerve (UN) on the contralateral side of the implanted S1. The first electrode was placed at the main branch of the UN, and the second at the dorsal cutaneous branch of the UN (DCBUN). We have no information on the proportion of sensory and motor fibres contained in these branches in pigs, but human studies suggest that the main branch of the UN is a mixed nerve, whereas the DCBUN is a purely sensory nerve [16]. Details about the implantation and characteristics of the peripheral nerve electrodes can be obtained from a previous study [17].

Electrical stimulation was delivered via a STG4008 programmable stimulator (Multichannel Systems, Reutlingen, Germany) at two stimulation intensities. The experimental protocol consisted of three stimulation trains separated by approximately 12 min. Each train comprised 50 stimulation pulses (amplitude = 1 mA, pulse duration = 500 μs) followed by 50 stimulation pulses to the DCBUN (amplitude = 5 mA, pulse duration = 1000 μs). Between each stimulation pulse, there was a 2 s delay with a pseudo-random Gaussian delay of ±250 ms to avoid habituation to repetitive stimulation.

A Tucker-Davis Technologies (TDT, Alachua, FL, USA) electrophysiology system was used to record brain signals. The signals were acquired through a low-insertion force head stage (ZIF-Clip) and connected to a battery-powered preamplifier (SI-8) that digitised the signals at a sample rate of 24 kHz. Signals were then streamed via a fibre optic connection to a data processor (RZ2), which received TTL pulses from the STG4008 to synchronise the stimulation and the recording systems. The recording system was configured and controlled using a computer workstation (WS8) via TDT’s Synapse software.

### 2.3. Signal Processing

MEA and uECoG signals were first windowed from −1.0 s to 1.5 s relative to the stimulation onset in each trial. The signals were then filtered using a fourth-order Butterworth design with half-power frequencies at 0.1 Hz and 5000 Hz. Further, a second-order Butterworth notch filter was applied to eliminate the 50 Hz line noise and its harmonics up to 400 Hz. All data analysis was performed using custom-made MATLAB algorithms (version R2023a, The Mathworks, Natick, MA, USA).

#### 2.3.1. SEP Amplitude and Signal-to-Noise Ratio

The peak-to-peak amplitude was measured from the SEPs in a 100 ms window from stimulation onset to capture the most prominent features of the SEPs (i.e., N1 and P1 peaks) [18]. The signal-to-noise ratio (SNR) was calculated by computing the ratio between the averaged peak-to-peak SEP amplitude in a window from the stimulation onset until 100 ms after stimulation to the root mean square of the baseline signal between 300 ms and 100 ms before stimulation onset. The SNR was converted to decibels defined as 20 × log10 (VppSEP/VrmsBaseline) and computed for somatosensory potentials evoked by the stimulation to the main branch of the UN and for stimulation to the DCBUN. Electrodes that did not display an identifiable ensemble average SEP were excluded from the analysis.

#### 2.3.2. Power Spectral Density

The power spectral density (PSD) was calculated at the channel and trial level for a period between 0 and 1 s after stimulation onset to obtain a higher frequency resolution. PSDs were estimated using a 1 s window with 50% overlap and a Hanning window. The PSDs were then averaged over channels and trials to obtain mean PSD estimates at the single-subject level. The PSDs were then split into the following frequency bands; 1–4 Hz (delta band, δ), 4–8 Hz (theta band, θ), 8–12 Hz (alpha band, α), 12–30 Hz (beta band, β), 30–80 Hz (gamma band, γ), 80–200 Hz (high-gamma band), 200–400 Hz (very high gamma band), 400–750 Hz, and 750–1500 Hz (multi-unit activity band, MUA) [19]. Finally, the power at each frequency band was compared between the MEA and the μECoG.

#### 2.3.3. Correlation Coefficient as a Function of Distance Between Electrode Pairs

Linear correlations were calculated for the trial-averaged responses considering a 1 s segment after stimulation onset using Pearson’s correlation coefficient. The correlation was estimated for all possible channel pairs, and pairs with the same Euclidean distances were pooled [20]. The absolute correlation values were considered since polarity inversion was observed for the SEPs recorded by the MEA.

### 2.4. Statistical Analysis

Data are presented as either boxplots or the mean ± standard error of the mean (s.e.m). A two-way repeated measures ANOVA was used considering the electrode-type (MEA vs. μECoG) as a between-subjects factor and nerve stimulated (UN vs. DBUN) as the within-subjects factor.

A significance value of 0.05 was adopted for all statistical tests, and the assumptions of homogeneity of variance and normality were assessed through residual analysis. Statistical analysis was performed in R version 4.3.3 [21].

## 3. Results

Two animals were excluded from the study because of surgical complications, noisy recordings, or noise contamination in the data collection, resulting in cortical activity severely affected by artefacts. Therefore, the following results represent a dataset from eighteen animals (μECoG = 10, MEA = 8).

### 3.1. SEP Amplitude and Signal-to-Noise Ratio

**Figure 2:**
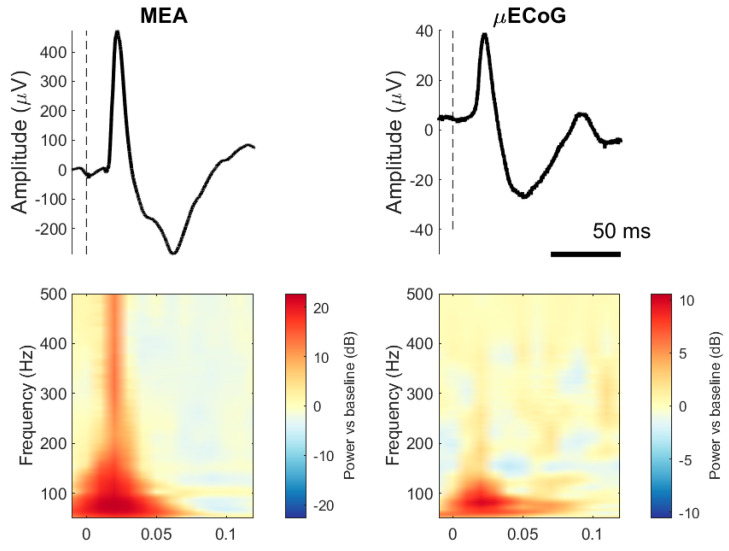
Somatosensory evoked potentials (SEPs) from two pigs, one recorded with an MEA and the other with an uECoG. (**Top row**): The line represents a trial-averaged single electrode (50 trials, one animal). Dashed lines indicate stimulation onset. (**Bottom row**): Indicates the time–frequency representation of the same SEPs with decibel normalisation.

Figure 2 (top) shows the ensembled average evoked potentials for one electrode and stimulation set (50 repetitions) recorded from the MEA and the μECoG and its respective spectrogram. Typical SEPs elicited by stimulation to the ulnar nerve for both interfaces are mainly characterised by two distinct components, P1 and N1, occurring between 20 and 60 ms after stimulation onset. It is noticeable from the figure that μECoG signals have a lower amplitude than MEA signals and that MEA contains more power at higher frequencies than μECoG SEPs.

**Figure 3:**
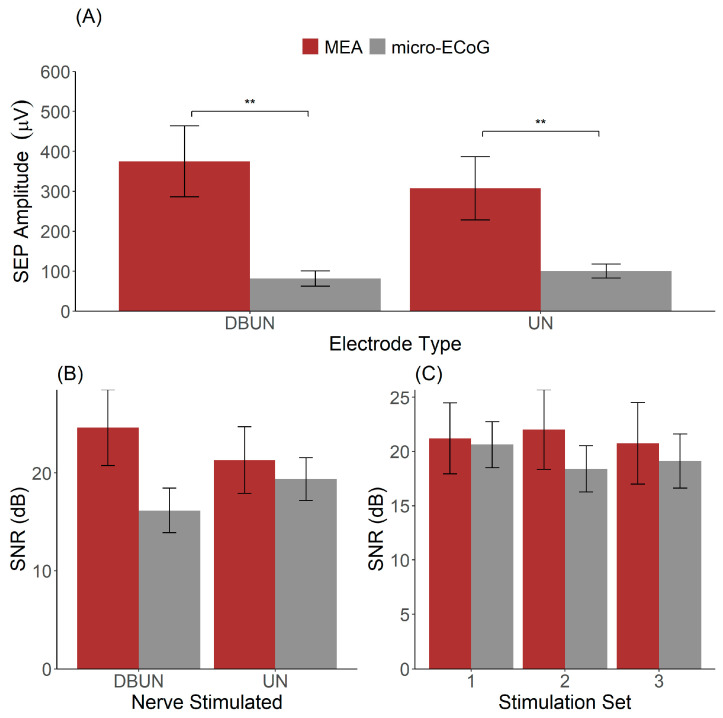
Recording quality of the μECoG and the MEA. (**A**) Distribution of SEP amplitudes across MEA and μECoG dependent on the nerve stimulated. (**B**) SNR as a function of electrode type and nerve stimulated. (**C**) Signal-to-noise ratio across stimulation sets presented 12 min apart for stimulation to the UN. ** indicates *p* < 0.01.

The peak-to-peak amplitudes of the SEPs were computed for all animals together with the signal-to-noise ratio, and the results are illustrated in Figure 3.

By analysing SEPs resulting from the stimulation to the DBUN and the UN, the results showed a significant effect of the interface type (MEA vs. µECoG, *p* = 0.01) and no significant effect of SEP amplitude based on the nerve stimulated (*p* = 0.99) (Figure 3A). SEPs recorded with the MEA had a higher amplitude than recordings with the μECoG for both stimulations to the DBUN.

Figure 3B shows the SNR for the two interfaces stimulated at the two distinct nerves. The two-way ANOVA resulted in a non-significant effect for both the electrode type on the SNR (*p* = 0.44) and for the stimulated nerve (*p* = 0.07). On average, the SNR obtained from SEPs resulting from stimulation to the UN was 21.3 ± 3.0 dB for the MEA versus 19.4 ± 1.9 dB for the μECoG. For SEPs resulting from stimulation to the DBUN, the SNR was 24.6 ± 3.4 for the MEA and 16.2 ± 2.0 for the μECoG.

Furthermore, we investigated the SNR across our three stimulation sets, as shown in Figure 3C. A two-way ANOVA with the animal as a within-subject factor and the interface type as a between-subject factor resulted in a non-significant main effect of interface type (*p* = 0.38) and stimulation set (*p* = 0.29). The averages across the stimulation sets 1, 2, and 3 were, respectively, 22.1 ± 3.3 dB, 23.3 ± 3.1 dB, and 22.0 ± 3.2 dB for the MEA and 19.8 ± 1.5 dB, 17.6 ± 2.1 dB, and 16.5 ± 2.2 dB for the μECoG. Although it reflects an acute experiment and stimulation sets 1 and 3 are separated by thirty minutes, Figure 3C shows that stable recordings were obtained.

### 3.2. Power Spectral Density

**Figure 4:**
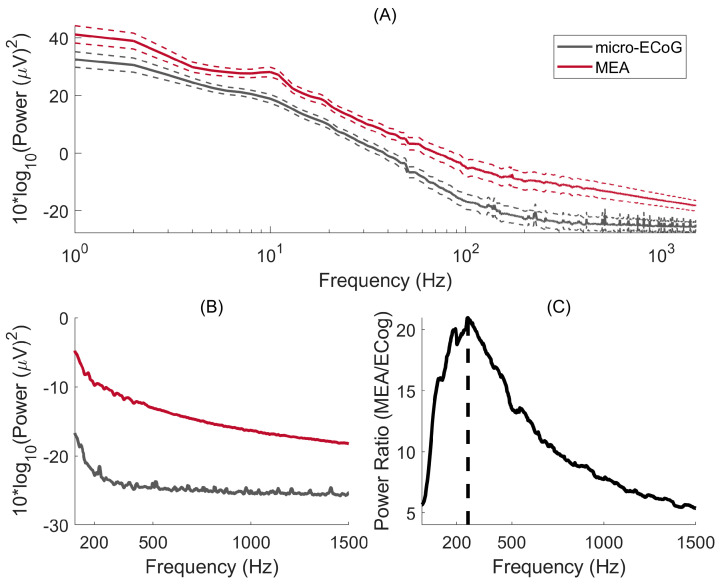
(**A**) Micro-ECoG and MEA power spectral density are plotted as a function of frequency. The continuous lines represent the median PSD, whereas the dashed lines indicate the standard error of the mean. (**B**) PSDs are represented on a linear scale. (**C**) Power ratio between MEA and micro-ECoG: the maximum power ratio occurs at a frequency of 264 Hz.

The power spectral density for signals recorded with μECoG and MEA are shown in Figure 4A. For both interfaces, significantly more power is obtained for the lower frequencies, as expected by the 1/f-like power spectrum of brain activity. Figure 4B shows the PSD on a linear scale where it becomes clearer that the μECoG PSD reaches a flat power spectrum for frequencies > 250 Hz. To explore this further, the ratio of power between MEA and μECoG is shown in Figure 4C, where one can observe that the maximum power ratio occurs at 261 Hz. As both PSDs decreased with an inverse power relationship, this result indicates that, at 261 Hz, the μECoG PSD slope stabilises, whereas MEA PSD continues to decline, resulting in a decreasing power ratio. The power ratio was six-fold for the lower frequencies (10 Hz) and peaked at 20.9 at 261 Hz.

The power obtained at the predefined frequency bands for the two interfaces and the power ratio between MEA and μECoG are displayed in Table 1.

### 3.3. Correlation of Electrode Pairs as a Function of Distance

**Figure 5:**
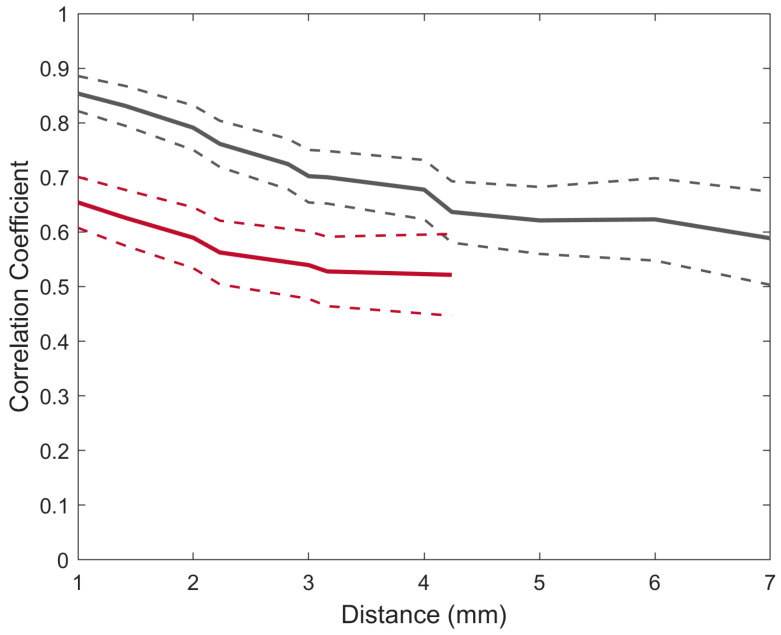
The average correlation between equidistant electrodes for broadband SEPs was recorded using a µECoG (grey) and an MEA (red). The dashed lines indicate the standard error of the mean.

The correlation values were initially estimated separately for SEPs evoked by stimulation to the motor branch and stimulation to the cutaneous branch; however, the correlation profile of both SEPs was highly similar. Therefore, the data were pooled to combine SEPs from stimulation from both nerves. The values of correlation of the SEPs between equidistant electrodes are displayed in Figure 5. Both the MEA and the µECoG had a centre-to-centre distance of 1 mm between the electrodes. The different distance scales are explained by the fact that the MEA contained 16 electrodes, whereas the µECoG contained 32 electrodes.

The results showed that both interfaces have a decreasing correlation as a function of distance. The highest correlation for both the MEA (*r* = 0.65) and the µECoG (*r* = 0.85) was at the 1 mm distance, and the lowest correlation coefficient for the MEA was at a distance of 4.24 mm (*r* = 0.52) and 7 mm for the µECoG (*r* = 0.55). A linear model was fitted for both correlation functions in order to estimate the slope of the correlation. The resulting slopes for the MEA and the µECoG were −0.1 and −0.09 per mm, respectively (*p* < 0.01).

## 4. Discussion

In the present study, we investigated somatosensory evoked potentials (SEPs) in the primary somatosensory cortex of pigs using an intracortical 16-electrodes MEA and a 32-electrode µECoG implanted subdurally. We aimed to characterise the commonly used features of SEPs (i.e., amplitude, SNR, PSD, and correlation structure) to investigate the difference between these two commonly used interfaces. SEPs were elicited by the stimulation of two distinct branches of the ulnar nerve. The clearest feature of the SEP morphology was a well-defined wave (P1, N1) with P1 already appearing at a latency of 20 ms, which was similar to a previous study using electrical stimulation applied to the finger of a macaque monkey and recording from the somatosensory cortex with an ECoG, where the authors reported the first SEPs at around 20 ms and termed this component short-latency SEP [22].

### 4.1. SEP Amplitude and SNR

SEPs recorded with an MEA had an overall higher peak-to-peak amplitude than µECoG SEPs, with an average ratio of 357%. The higher SEP amplitude was the case for both stimulation to the main branch of the UN and the DBUN. Obtaining a higher SEP amplitude with intracortical electrodes is an expected result, as the two major sink–source complexes of SEPs are in supragranular layers (I–IV) and infragranular layers (V–VI) [23]. Previously, in a reach-and-grasp task, researchers have shown that the amplitude similarity between intracortical LFPs and ECoGs from the primary motor cortex decreases as a function of cortical depth, which is accentuated for high-frequency components such as high-gamma [9]. The same effect was observed in another study assessing auditory evoked potentials [24]. In the latter, contrary to our results, the amplitude of evoked responses was significantly higher for the ECoG than those recorded with intracortical electrodes, which might be explained by the significant difference in impedances or the larger surface area.

In order to measure the relation between the signal (SEPs) and “noise”, we calculated a signal-to-noise ratio (SNR) as the ratio of the amplitude of the peak-to-peak SEPs and the root mean square of spontaneous brain activity. Interestingly, no significant differences in SNR were observed between MEA and µECoG SEPs. However, one can argue that a trend exists since average SNR values were higher for both UN and DBUN stimulations. The absence of SNR differences from these interfaces indicates a higher amplitude in the baseline activity recorded by the MEA. Still, this result is highly promising as higher SNRs increase the capability of a system to decode a task-related event and have direct implications for intraoperative monitoring, where reliable SEP estimates can be obtained with fewer trials. Nevertheless, since the SEPs were elicited by direct nerve electrical stimulation, the SNR values were expected to be relatively high. Electrical stimulation is a non-natural stimulus and activates nerve fibres in an inverse recruitment order (i.e., increasing the current first activates the large fibres and then progresses to the smaller fibres); consequently, SEP values obtained by the natural stimulus are likely to result in lower SNR values.

### 4.2. Comparison of Power Spectral Density

The power spectral density of brain signals is most well characterised for frequencies up to 100 Hz because of the widespread use of EEG systems. However, there is a growing interest in high-frequency oscillations as a mechanism of neuronal communication [25] that can serve as a biomarker in, e.g., the localisation of epileptogenic zones [26]. While penetrating electrodes can record broadband signals from LFPs (<300 Hz) generated by synchronised postsynaptic potentials and multi-unit activity from action potentials (0.1–10 kHz) [27], signals recorded with µECoG have a more limited frequency range. In our study, using a µECoG with an electrode diameter of 200 μm, the power ratio of MEA/µECoG has an average of 5.5 in frequencies below 30 Hz and 9.9 in the gamma range. This ratio continued to increase as a function of frequency, reaching its peak of 20.9 at 261 Hz and having an average of 18.3 in the high gamma range (80–200 Hz) and very-high gamma range (200–400 Hz). To put this into perspective, the ratio between ECoG power and EEG power in frequencies up to 56 Hz is, on average, 16-fold [28].

Interestingly, a previous study in humans found that the brain’s surface electric potential contains a distinct power-law scaling between low frequencies (*f* < 80 Hz) and higher frequencies (80 Hz < *f* < noise floor) [29]. Our results suggest that this is also the case also for the pig’s cerebral cortex, given that the power ratio is, on average, 6.4 at *f* < 80 Hz and 17. 3 at 80 Hz < *f* < 400 Hz. However, we can only speculate that this different power-law form is also present here, given that the empirical noise floor was not measured. Thus, we can only have an estimation of the noise floor by assessing the frequency at which the PSD slope becomes flat and through the power ratio, which in both cases indicates a value of *f* > 250 Hz.

### 4.3. Correlation of Electrode Pairs as a Function of Distance

We quantified the similarity of the broadband SEPs via correlation analysis across pairs of electrodes separated by the same Euclidean distances. Mapping the correlation of cortical activity across all the electrodes indicates the amount of spatially independent information that can be obtained from these recordings, and it has direct implications for an optimal design of the electrode, where information has to be maximised and distributed to a limited number of electrodes. Naturally, the spatial correlation of brain activity not only depends on electrode geometry but also on the task performed, brain areas, and cortical anatomy [30]. However, several studies across different species and tasks demonstrated that the correlation of neural activity is inversely related to the distance between electrodes [7,20,31,32]. For both interfaces, it was observed that the spatial correlation of SEPs was inversely related to the distance and that the correlation was higher for µECoG SEPs than SEPs recorded with the MEA, suggesting that MEAs contain a higher spatial resolution than µECoG grids. One important point to consider is that the correlation functions were mapped for SEPs elicited by electrical stimulation, which is non-specific, meaning that, at high levels of current, it simultaneously activates the neurons responsible for conveying information from several sensory categories, which can lead to widespread spatial patterns that correlate with stimulation intensity [22]; therefore, it can be expected that the correlation between electrodes would be lower (i.e., less redundant information) for tasks that involve naturalistic stimuli. Finally, this study focused on broadband SEPs and correlation versus distance at distinct frequency bands was not assessed. However, the evidence shows a frequency dependence of spatial correlation, where low frequencies are more widespread than higher frequencies [20,31].

### 4.4. Limitations

A limitation of our study is the use of separate cohorts for MEA and µECoG recordings. The simultaneous implantation of both electrode modalities in the same animals has been previously performed with custom-made electrodes [33] and can minimise inter-subject variability. However, by using electrical stimulation to evoke SEPs, we ensured a reliable and reproducible input across all animals, which minimises the variability that could arise from natural sensory inputs or spontaneous neural activity. In addition, the electrical stimulation amplitude was set well above motor thresholds, ensuring that the activation of a substantial proportion of peripheral nerve fibres, dominating the neural responses, and making the outcomes predominantly driven by the experimental stimulation.

Our study investigated SEPs with frequencies up to the multi-unit activity (MUA) level. However, prior research has shown that µECoG arrays are capable of extracting single-unit spikes [33] and that single-unit activity recorded from µECoG arrays can be used to decode the spatial position during free navigation [34]. In both cited studies, the electrode’s surface area was considerably smaller than those in our µECoG arrays, and modelling studies suggest that smaller surface areas are more effective for capturing single-unit activity [35].

## 5. Conclusion

This study explored the main signal characteristics of µECoG and penetrating MEAs using somatosensory evoked potentials recorded from the pig’s primary somatosensory cortex. Taken together, significantly higher amplitudes were observed from MEA signals, while SNR was similar across both interfaces. Power differences were more pronounced for high-gamma ranges (> 80 Hz), and correlation as a function of distance decreased at the same rate for both interfaces; however, slightly higher correlations were observed for the µECoG.

## Figures and Tables

**Table 1:** Group-averaged power (dB) was obtained by recording broadband SEPs in the primary somatosensory cortex with an μECoG and an MEA at commonly adopted frequency bands. The fourth column indicates the raw power ratio between the MEA and the μECoG. * indicates *p* < 0.05.

Frequency	MEA	μECoG	Power Ratio
δ: 1–4	35.1 ± 2.8	28.4 ± 2.3	5.2
θ: 4–8	28.7 ± 2.2	21.8 ± 1.3	4.6
α: 8–12	26.4 ± 2.1	18.2 ± 1.4	7.2
β: 12–30	16.1 ± 2.2	9.0 ± 1.8	5.3
γ: 30–80	−4.9 ± 2.3	0.7 ± 2.0	9.9
High γ: 80–200	−6.5 ± 1.1	−15.2 ± 2.6	17.5
Very high γ: 200–400	−10.7 ± 0.6	−18.7 ± 2.5	19.2
MUA: 400–750	−13.5 ± 0.5	−20.1 ± 2.2	12.9
MUA: 750–1500 *	−16.6 ± 0.4	−21.8 ± 1.8	7.2

## Data Availability

Data from this study are available from the corresponding author, F.R.A., upon reasonable request.
